# Exploring pain and suffering through spatial acousmatic music: innovative perspectives beyond conventional music therapy

**DOI:** 10.3389/fpain.2025.1672412

**Published:** 2025-09-26

**Authors:** Nikos Stavropoulos, Mark I. Johnson

**Affiliations:** ^1^ School of Arts, Leeds Beckett University, Leeds, United Kingdom; ^2^ Centre for Pain Research, School of Health, Leeds Beckett University, Leeds, United Kingdom

**Keywords:** pain, acousmatic music, music therapy, sound, suffering, emotional memory

## Abstract

In this perspective article we contend that acousmatic music, which departs from the traditional “instrumental music paradigm” by obscuring or removing the origin of sounds, may deepen a person's understanding and expression of pain and suffering, offering therapeutic potential. We propose that intentional engagement with acousmatic music can reshape listening habits, articulate and reframe the meaning of bodily and emotional experiences, and alleviate distressing sensations, feelings and thoughts. We propose that acousmatic music evokes memories of previous psychological traumas, such as painful events, and by doing so can prompt listeners to curiously explore the meaning and purpose of distressing symptoms. We argue that creative engagement with acousmatic music may allow individuals to express their somatic, emotional, and cognitive experiences, potentially leading to a deeper understanding of their living experiences. We discuss future directions for research and practice. We offer readers a stereo reduction excerpt of acousmatic music to facilitate an appreciation of the unusual nature of acousmatic music composition (https://soundcloud.com/nikos-stavropoulos/topophilia).

## Introduction

There is growing use of engagement with art, including music, to alleviate suffering and facilitate “healing” of various health conditions reflected in a proliferation of systematic reviews and meta-analyses evaluating the impact of music for therapy, for example in the management of pain [e.g., the following since 2022 (1–17)]. Research on the use of music in healthcare settings focuses on the “instrumental music paradigm,” based on the concept of musical notes; an arrangement of sound(s) that creates expressive content through combinations of form, harmony, melody, and rhythm of determinate pitched sounds of a certain stability and duration (18)—what the general public may recognise as familiar, conventional music.

The instrumental music paradigm is an oversimplification of the nature of music and treats music as a homogeneous “intervention”, overlooking the inherent complexity and diversity within and between musical genres and neglecting influence of the characteristics within and between specific pieces of music (19, 20). Martin-Saavedra and Saade-Lemus (21) advocate for a more objective analysis of music, employing theoretical terms such as mode and tempo, rather than relying solely on genre or artist. This broader analytical framework may reveal new avenues for exploring music's therapeutic potential. For instance, a preclinical experimental study in mice by Zhou et al. (22) demonstrated that the signal-to-noise ratio—the volume of sound relative to ambient noise—is critical in raising nociceptive thresholds, indicating that the delivery and environmental context of sound significantly influence nociceptive responses. Their findings revealed that even simple sounds, such as white noise, can modulate the flow of nociceptive information through corticothalamic circuits. This challenges the assumption that only structured or traditionally “musical” sounds possess therapeutic potential and highlights the role of contextual factors in music-induced analgesia. It also opens the door to broader, more accessible sound-based interventions in healthcare.

The aim of this article is to present a perspective on the potential of acousmatic music to express and deepen an individual's understanding of their lived experiences of pain and suffering through spatial sound, moving beyond the scope of conventional music therapy.

## Perspective

We contend that acousmatic music—by obscuring or removing the source of sound and departing from the traditional instrumental music paradigm—may deepen a person's understanding and expression of pain and suffering, offering therapeutic potential.

Our perspective has been shaped in part by informal conversations with audiences comprising individuals both with and without lived experience of pain, following short acousmatic music performances. Many audience members were surprised by how acousmatic music sparked emotional curiosity—particularly around experiences of pain—in ways that differed from more familiar, conventional music. This led us to consider whether acousmatic music might support deeper reflection on health and illness. We suggest that it can foster emotional insight, openness, and empathy by encouraging listeners to explore their own feelings, engage with a wide range of emotions, and consider the experiences of others. This process may offer new ways of understanding and easing suffering, while supporting a more holistic and personally meaningful approach to healing.

Our discussion is grounded in our interdisciplinary perspectives as an artist (NS, Professor of Music Composition) and a pain scientist (MIJ, Professor of Pain and Analgesia), drawing on our combined expertise to explore how acousmatic sound may open new avenues for engaging with health, illness, and healing. Acousmatic music is a genre characterized by the deliberate obscuring of sound sources, thereby encouraging listeners to focus exclusively on auditory qualities. Explaining the nature of acousmatic music in words undersells the listening experience so we offer some examples here and encourage readers to listen before proceeding (https://soundcloud.com/nikos-stavropoulos/topophilia).

## Discussion

### Theoretical foundations

Acousmatic music diverges from the traditional instrumental music paradigm by deliberately obscuring the origin of sounds, thereby encouraging a focus on the auditory experience itself. The term “acousmatic” originates from the teachings of Pythagoras, who delivered lectures from behind a screen to ensure that his students concentrated solely on his voice (23). In contemporary practice, acousmatic music is a genre of electronic music that emphasizes the separation of sound materials from their original context, offering a unique listening experience that is both immersive and abstract.

Acousmatic music is distinguished by source ambiguity, which intentionally obscures or removes the origin of sounds, thereby fostering a purely auditory engagement (24).

“…electroacoustic (is) more concerned with spectral qualities than actual notes, more concerned with varieties of motion and flexible fluctuations in time rather than metrical time, more concerned to account for sounds whose sources and causes are relatively mysterious or ambiguous rather than blatantly obvious.” (24) p. 109.

Acousmatic music often exploits the dual nature of sound as both an aural phenomenon and a sign or referent, recontextualizing real-world sounds within new musical frameworks (25).

The absence of visual stimuli develops and strengthens cognitive and sensory aspects of listening. This allows listeners to engage with and interpret abstract soundscapes, constructing meaning, mental images, and narratives on auditory stimuli alone. Acousmatic music often confronts listeners with unfamiliar aural experiences—both in the content of the sound itself but also in obscuring the sound sources—inviting them to question perceptual habits and engage with new ways of interpreting sound phenomena (26).

Beard (26) describes how acousmatic listening can be used to disrupt habitual listening patterns *“…as an intentional practice, one that will enable critical reflection on listening”* (26) p. 129. This intentional practice fosters reflection and contemplation with potential to interrupt listening-habits, opening a different listening experience.

“When we stop trying to decide whether we recognize a sound, or whether we can imagine the source of a sound, and instead immerse ourselves in the experience of the sound, we listen differently.” (26) p. 131

### Proposed mechanisms

Acousmatic music encourages engagement with multiple listening modes including (27):

• **Reduced Listening:** Focusing on the intrinsic features of sound, independent of its source.
• **Causal Listening:** Speculating about the potential sources of sounds.
• **Semantic Listening:** Interpreting symbolic meanings or personal associations with sounds.
• **Rhetorical Listening:** Analysing how sounds relate to each other within the composition's narrative or emotional arc.

Acousmatic listening involves focussing on the sound itself and making elements of the listening experience explicit, rather than trying to recognise the sound or querying its origin. This requires an “acousmatic attitude” (28) p. 5 that shifts a listening activity from being reflexive to reflective by,

“…strip[ping] away expectations and schema that interfere with the listening process” (26) p. 131.

Kane's ontology of the acousmatic experience (29), highlights the significance of the empty space between the source, cause, and effect of acousmatic sound,

“ …the being of acousmatic sound is to be a gap… ” (29) p149.

Soddell (30) identifies the potential of this gap to facilitate free connections between sounds, real-world events, lived experiences and more abstract concepts and exploit the acousmatic experience to “understand states of anxiety and distress, and the inner world of the psyche”, using what Soddell terms as *Experiential Listening* as a method for shaping and understanding ideas through sound (30).

Spatialization and sound movement are integral aspects of acousmatic music, enhancing sensory experiences and deepening emotional engagement (31). The immersive nature of acousmatic music facilitates a heightened sense of presence, encouraging greater cognitive focus and emotional responses to auditory stimuli. Furthermore, acousmatic music has the potential to facilitate communication and social interaction by reappraising sensory input and developing cognitive control strategies (32). Techniques employed in acousmatic music can evoke powerful emotional responses, drawing upon and expanding real-life experiences (33, 34).

### Therapeutic potential

The therapeutic potential of acousmatic music remains largely unexplored, and a search of PubMed on 08 April 2024 using the search term “pain” combined with “acousmatic” or “music” or “sound” or “audio” failed to identify any records pertaining to acousmatic music (Supplementary Material). To our knowledge there has been no attempt to explore the potential of acousmatic sound for health and wellbeing.

**Figure 1:**
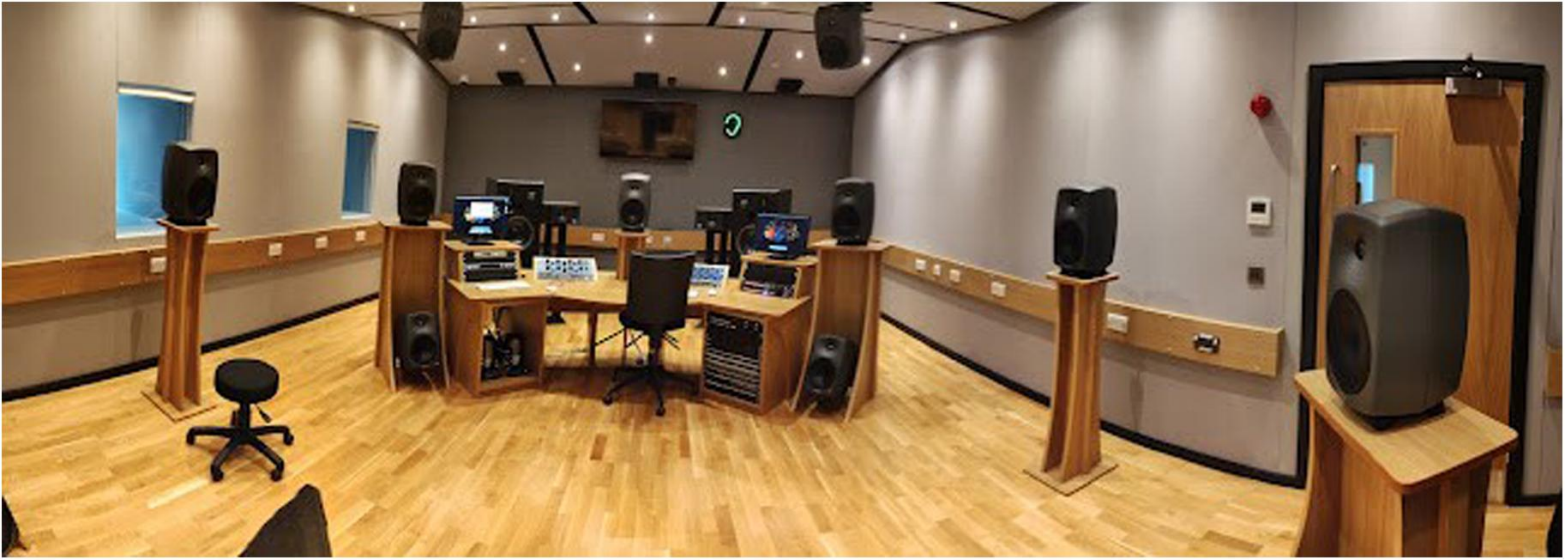
The periphonic (full 3D) ambisonics studio at Leeds Beckett University.

Based on our experiences delivering short, 15-min performances of acousmatic music to academic colleagues, and to a group of cancer sufferers and survivors—both with and without pain—in our periphonic (full 3D) Ambisonics studio (Figure 1), we have found that audiences are often surprised by the nature of acousmatic music. It is something they have never encountered before. Performances comprise two immersive acousmatic works by Stavropoulos, Topophilia and Khemenu that explore the notion of aural intimacy through novel recording techniques, using bespoke microphone hardware, and new ways of using ambisonics technology in the composition of acousmatic works (https://soundcloud.com/nikos-stavropoulos/topophilia) (33). Both compositions display strong spatiality, increased perceived materiality of sound materials and are acknowledged internationally in various composition competitions.

Acousmatic music seems to trigger emotional responses, especially related to previous trauma and adversity such as painfully distressing medical procedures and events. Emotional memories seem to be both “negative”, (e.g., threat, anxiety, goosebumps, increased somatosensory awareness of aches and pains) and “positive” (e.g., sense of calm, comfort, happiness, and bodily warmth). We are interested in how elements within the music trigger emotional memories and whether acousmatic music practice could be used to spark conversations and curious exploration of illness, health and well-being.

By engaging several listening modes simultaneously, acousmatic music connects the cognitive aspects of musical experience to the physiological response(s) to auditory stimuli. This connection is enhanced by the construction and articulation of acoustic space, which is often an integral part of acousmatic music practice. Immersive sound experiences contribute to a person's sense of presence and engagement, which enhances cognitive focus and emotional responses to [the acousmatic] stimuli. Moreover, acousmatic approaches engage spatial auditory perception either by recreating real auditory spaces, enhancing the real experience of those for the listener by augmenting or subverting those, as well as creating hyperreal or imaginary acoustic spaces.

#### Acousmatic music and pain: toward a therapeutic framework

To further investigate the therapeutic potential of acousmatic music, we propose four lines of inquiry:

1. Reshaping the listening experience

From our perspective, acousmatic music may foster the development of new listening skills, enabling individuals to “filter in” soothing sounds while “filtering out” distressing sounds associated with experiences such as pain. Audience members report that “unnatural” or “threatening” sounds—such as metal-on-metal, scream-like tones, or overbearing noise—often triggered unpleasant memories and emotions. In contrast, “natural” or “safety” sounds—such as nature-like textures, welcoming tones, and soothing rhythms—evoked comforting and pleasant associations. This suggests a potential for acousmatic music to support the re-association of sounds linked to painful experiences with more positive emotional responses. As a distinct and intentional listening experience, acousmatic music opens new listening channels and cultivates the ability to engage with sound in a more exploratory and therapeutic way.

2. Exploring sounds from the past

From our perspective, acousmatic music has the potential to trigger latent emotional memories, facilitating a journey of discovery and recovery. This form of listening may be particularly valuable in contexts where trauma—whether major (“big T”) or minor (“little t”), including adverse medical procedures or childhood experiences—has contributed to the persistence of pain and suffering. Acousmatic music offers a means of bringing non-conscious emotional memories into awareness to prompt reflection, discussion, and curious exploration of past, present, and future experiences of living with pain. When guided appropriately by healthcare professionals or mind-health coaches, this process may support reconceptualization of pain-related experiences and emotional healing.

3. Expressing painful experiences

There is strong evidence that people often struggle to express their pain to others, and even when they do, they may feel unheard (35). From our perspective, acousmatic music production offers a creative and empowering avenue for individuals living with pain to articulate their experiences. Using simple recording devices—such as smartphones—and basic, easily learned music engineering techniques, individuals can capture sound bites and compose musical representations of their pain. With support from a music technician if needed, these compositions can serve as powerful tools for expressing distressing sensory, emotional, or cognitive experiences. Sharing such works with healthcare professionals during clinical consultations may enhance communication, foster empathy, and support more person-centred care. This process not only validates the lived experience of pain but also opens new possibilities for therapeutic engagement through sound.

4. Reshaping the meaning of pain

From our perspective, acousmatic music may support individuals in reconceptualizing the meaning of distressing sensory, emotional, and cognitive experiences, moving beyond simplistic narratives—such as the belief that pain necessarily indicates bodily damage. Many people struggle to make sense of their pain when biomedical treatments fail, often finding it difficult to attribute any positive or meaningful interpretation to their suffering (36). Engagement with the arts can help individuals reframe the meaning of pain by fostering a more holistic understanding of the embodied self, situated within a broader social and emotional context (37). Acousmatic music, in particular, offers a unique tool for exploring, expressing, and reshaping the meaning of lived experiences, including pain. By matching sound characteristics—such as texture, rhythm, timing, timbre, and pitch—to sensations, emotions, and thoughts, individuals can create sonic representations of their experiences. This process might include mapping pain-event timelines, expressing the rhythm of a pain journey, or exploring themes such as grief, growth, and transformation. Through this creative engagement, acousmatic music may open new pathways for understanding and healing.

## Risks, challenges, and ethical considerations

The abstract, non-representational soundscapes of acousmatic music present risks, challenges, and ethical considerations that must be navigated to ensure safe and inclusive practice.

### Individual variability in perception and response

The degree of individual variability in how people living with pain perceive and emotionally respond to acousmatic music is unknown. Responses to acousmatic music are likely shaped by personal auditory associations, neurological sensitivity, cultural background, and cognitive readiness. Hence, acousmatic music may liberate imaginative engagement and facilitate cognitive distraction from pain in some individuals, but the lack of familiar musical cues may also provoke confusion, discomfort, or emotional disengagement in others.

Ethical practice necessitates a highly individualized approach that involves obtaining informed consent that clearly explains the nature of acousmatic music and its potential effects. Collaborative music selection should be encouraged, allowing participants to choose or reject sound materials based on their comfort and preferences. Cultural sensitivity is also essential, as abstract sounds may carry unintended symbolic or emotional weight depending on the listener's background.

### Emotional distress and trauma sensitivity

Acousmatic music has potential to evoke deep emotional responses. Certain sonic textures—such as dissonant frequencies, distorted voices, or abrupt dynamic shifts—may inadvertently trigger traumatic memories or psychological distress, particularly in individuals with a history of post-traumatic stress, anxiety, or depression. This risk is pertinent in people with persistent pain where co-occurring mental health conditions are common.

Ethical practice necessitates screening for trauma history and emotional sensitivity prior to introducing acousmatic sound. Sessions should be structured to avoid coercion or pressure to engage with soundscapes that provoke discomfort and emotional responses closely monitored with provision for immediate support and opt-out mechanisms if distress occurs. Facilitators must be trained to recognize signs of emotional flooding and respond with appropriate interventions, including grounding techniques and referral to psychological support when necessary.

### Sensory overload and cognitive fatigue

The complex, layered, and unpredictable nature of acousmatic music poses a risk of sensory overstimulation, particularly for individuals with heightened sensory sensitivity—a common feature for people living with pain. The cognitive effort required to interpret and make sense of abstract soundscapes may lead to mental fatigue, exacerbating symptoms such as “brain fog” or reduced cognitive capacity due to pain or medication.

To mitigate these risks, facilitators should carefully curate the listening experience, selecting sound materials that are appropriate to the sensory profile and cognitive state of participants. Session pacing should be adjusted to avoid overstimulation, and breaks should be incorporated to allow for cognitive recovery. Ethical practice involves educating participants about the potential for sensory overload and encouraging open communication about their experiences during and after sessions.

### Therapeutic accessibility and engagement

Acousmatic music is unfamiliar to most people and individuals may find the genre alienating or difficult to engage with meaningfully and facilitators should introduce and scaffold the listening experience in a way that fosters openness and emotional resonance by demystifying the genre through clear explanations and examples that emphasizes therapeutic potential rather than artistic complexity. Participants should be empowered to actively engage in the listening process, including the option to reject or modify sound materials that do not resonate with them. Therapeutic opportunities should not be limited by technical knowledge or cultural familiarity but focus on creating inclusive, person-centred experiences that respect individual preferences and needs.

### Specialized expertise and safe facilitation

The safe and effective use of acousmatic music in therapeutic contexts requires facilitators—whether artists or therapists—to possess foundational digital audio skills and a working knowledge of sound design. These competencies are essential not only for adapting the listening experience to meet individual needs, but also for guiding participants through abstract emotional landscapes in a way that facilitates reflective dialogue and allows for close monitoring of emotional distress.

When participants are encouraged to compose or manipulate sound, the process must be ethically structured through supportive guidance that establishes clear boundaries around emotional content and sound choices. To ensure emotional safety and therapeutic coherence, debriefing sessions should be incorporated to help participants process both their creative output and any emotional reactions that may arise. Ethical practice demands that the therapeutic process remain coherent, responsive, and attuned to the participant's emotional state throughout.

### Misconceptions around technical requirements

A common misconception is that acousmatic music therapy demands advanced compositional expertise and professional studio facilities. While high-fidelity listening environments can enhance the experience, basic tools such as stereo speakers, headphones, and mobile phones are sufficient for therapeutic engagement. Accessible software platforms allow for sound manipulation without requiring extensive technical training.

Ethically, facilitators should emphasize the simplicity and accessibility of the process, empowering patients to participate in sound creation using everyday technology. This democratization of sound creation not only reduces barriers to participation but also enhances the therapeutic value by fostering agency, creativity, and emotional expression. Facilitators should avoid perpetuating the notion that acousmatic therapy is reserved for those with specialized skills or resources, and instead promote inclusive practices that prioritize participant empowerment.

### Integration with broader care plans

In healthcare settings, acousmatic music therapy should not be viewed as a standalone intervention but rather as a complementary component within a broader pain management strategy. Pain specialists must avoid over-reliance on music therapy as a singular solution and instead position it within a multidisciplinary framework that includes pharmacological, psychological, and physical interventions.

Ethical integration involves ongoing dialogue with patients about their goals, preferences, and progress, fostering a collaborative and transparent therapeutic relationship. Ethical practice involves coordination with multidisciplinary pain teams, including psychological professionals to ensure compatibility and continuity of care. Documentation and evaluation of therapeutic outcomes through records of patient responses, session content, and any adverse reactions, are essential to ensure efficacy and safety and to refine therapeutic approaches.

## Future research

While we did not collect qualitative data from audiences following the performances—a limitation we fully acknowledge—this perspective paper aims to share our initial observations and signal the need for further empirical investigation. An obvious and essential first step is to capture the phenomenology of engaging with acousmatic music among people living with pain. To this end, we have already designed an interdisciplinary study exploring how acousmatic music can facilitate connections between memories, events, thoughts, and emotions. Using a participatory approach, the study will engage participants in listening, co-creation, and active dialogue through a series of structured activities. An embedded qualitative component will be used to better understand the impact of the intervention. The research team will bring together expertise in acousmatic music and health sciences from Leeds Beckett University, in collaboration with Balbir Singh Dance Company—an Arts Council National Portfolio Organisation known for its innovative community engagement—and community-based networks such as the Mid Yorkshire Breast Cancer Support Group. This phenomenological approach would help ground future experimental and clinical research in the realities of those who may benefit most.

The lack of experimental research on acousmatic music presents significant opportunities to investigate its effects on psychophysiological responses, particularly in relation to pain and health. This includes exploring how compositional structures, sound intensity levels, and spatial auditory perceptions influence physiological and emotional states. As a foundational step, we propose a scoping review to map the current state of knowledge and practice concerning acousmatic music and health. Experimental approaches could then examine responses in diverse populations, such as individuals living with persistent pain or healthy participants exposed to noxious stimuli. Variables of interest might include compositional structures (e.g., texture-carried, gesture-carried, or hybrid forms), stable vs. unstable sound spectra, event rate, musical form, and intensity dynamics. Further dimensions include the use of relaxation- vs. stress-inducing materials (e.g., expectation, release, resolution, stasis), spatial disposition and trajectory, sensory deprivation to heighten cognitive focus, recognition of sound source and cause, spectromorphological and spatiomorphological change, and auditory immersion formats (e.g., binaural, discrete channels, Higher Order Ambisonics). These parameters offer a rich terrain for investigating how acousmatic music may modulate perception, emotion, and bodily experience.

## Conclusion

We contend that the intentional practice of acousmatic music, whether through listening or production, presents promising opportunities for enhancing health and wellbeing, particularly in contexts where people experience long-standing symptoms causing distress and suffering, e.g., persistent pain. Acousmatic music, by embedding listeners in unfamiliar auditory environments, offers unique opportunities for engaging with sensory, emotional, and cognitive reactions that may lead to new understandings and therapeutic strategies, including opening up conversations with others. We intend to direct future research on elucidating the potential of acousmatic music to reshape listening, articulate living experiences and evoke memories, thereby facilitating a deeper exploration of suffering. Art-led practices, including engagement with music, are increasingly recognized as effective modalities for supporting individuals living with pain and other long-term conditions e.g., the Unmasking Pain project (37, 38). Future collaborations with arts organizations could facilitate the development and evaluation of how acousmatic music may be utilized to express and explore the narratives of living with pain.

## Data Availability

The original contributions presented in the study are included in the article/Supplementary Material, further inquiries can be directed to the corresponding author.
