# Supplementary material for: Exploring pain and suffering through spatial acousmatic music: innovative perspectives beyond conventional music therapy
Source: Front Pain Res (Lausanne). 2025 Sep 26;6:1672412. doi: 10.3389/fpain.2025.1672412 (PMC12511085; doi:10.3389/fpain.2025.1672412)
Supplement: Supplementary file 1 [file Table1.docx]

Supplementary Material

Search Terms

Sound

- Acousmatic (sound that is heard without an originating cause being seen)
- Acousmatic Music
- Acousmatic Listening
- Electroacoustic Music
- Soundscape / Soundscape Composition
- Immersive Audio / Spatial Audio
- Reduced Listening
- Schaefferian Listening Modes
- Ambisonics (Spatial Audio Technique)
- Spectromorphology

Pain

- Pain
- Analgesia

Search Strategy

PubMed: 21 December 2023 and updated on 08 April 2024 [All Fields] – [Search strings here](https://pubmed.ncbi.nlm.nih.gov/advanced/)

Findings

[Pain AND music](https://pubmed.ncbi.nlm.nih.gov/?term=%28pain%29+AND+%28music%29&sort=date) = 2213 – [224 SRs](https://pubmed.ncbi.nlm.nih.gov/?term=%28pain%29+AND+%28music%29&filter=pubt.systematicreview&sort=date&size=200) – music for procedural pains

[Pain AND sound](https://pubmed.ncbi.nlm.nih.gov/?term=%28pain%29+AND+%28sound%29&sort=date&size=200) = 6153 – [256 SRs](https://pubmed.ncbi.nlm.nih.gov/?term=%28pain%29+AND+%28sound%29&filter=pubt.systematicreview&sort=date&size=200)

[Pain AND audio](https://pubmed.ncbi.nlm.nih.gov/?term=%28pain%29+AND+%28audio%29&sort=date&size=200) = 1185 – [33 SRs](https://pubmed.ncbi.nlm.nih.gov/?term=%28pain%29+AND+%28audio%29&filter=pubt.systematicreview&sort=date&size=200) – audio-visual distraction

Pain AND acousmatic = 0

[Pain AND Electroacoustic](https://pubmed.ncbi.nlm.nih.gov/34487218/) = 1 – not relevant

[Pain AND Soundscape](https://pubmed.ncbi.nlm.nih.gov/?term=soundscape+AND+pain&sort=date&size=200) = 3 – not relevant

[Pain AND immersive audio](https://pubmed.ncbi.nlm.nih.gov/?term=%28Immersive+Audio%29+AND+%28pain%29&sort=date) = 14 – – not relevant - mostly VR/AR and multimodal (visual)

[Pain AND spatial audio](https://pubmed.ncbi.nlm.nih.gov/?term=%28Spatial+Audio%29+AND+%28pain%29&sort=date) = 5 – not relevant

[Pain AND reduced listening](https://pubmed.ncbi.nlm.nih.gov/?term=%28Reduced+Listening%29+AND+%28pain%29&sort=date) = 498 – mostly ‘listening’ to music ‘reduced’ pain – probably captured in music search

[Pain AND Ambisonics](https://pubmed.ncbi.nlm.nih.gov/?term=Ambisonics+AND+pain&sort=date&size=200) = 0

Pain AND Spectromorphology = 0

Acousmatic <https://pubmed.ncbi.nlm.nih.gov/?term=Acousmatic&sort=date>

Electroacoustic - <https://pubmed.ncbi.nlm.nih.gov/?term=Electroacoustic&sort=date>

Electroacoustic Music - <https://pubmed.ncbi.nlm.nih.gov/?term=Electroacoustic+music&sort=date>

Soundscape - <https://pubmed.ncbi.nlm.nih.gov/?term=soundscape&sort=date>

Soundscape Composition - <https://pubmed.ncbi.nlm.nih.gov/?term=Soundscape+Composition&sort=date>

Immersive Audio - <https://pubmed.ncbi.nlm.nih.gov/?term=Immersive+Audio&sort=date>

Spatial Audio - <https://pubmed.ncbi.nlm.nih.gov/?term=Spatial+Audio&sort=date>

Reduced Listening - <https://pubmed.ncbi.nlm.nih.gov/?term=Reduced+Listening&sort=date>

Schaefferian Listening Modes - 0

Ambisonics (Spatial Audio Technique) - <https://pubmed.ncbi.nlm.nih.gov/?term=Ambisonics&sort=date>

Spectromorphology - 0
